# Supplementary material for: The RNA quality control pathway nonsense-mediated mRNA decay targets cellular and viral RNAs to restrict KSHV
Source: Nat Commun. 2020 Jul 3;11:3345. doi: 10.1038/s41467-020-17151-2 (PMC7334219; doi:10.1038/s41467-020-17151-2)
Supplement: Supplementary file 1 — Supplementary Information [file 41467_2020_17151_MOESM1_ESM.pdf]

Supplementary Information

The RNA quality control pathway nonsense-mediated mRNA decay targets cellular and viral RNAs to restrict KSHV

Zhao et al.

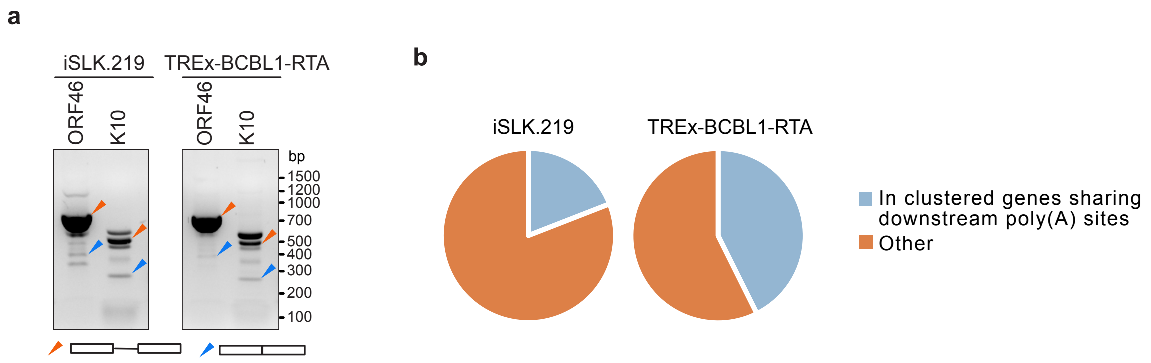

Supplementary Figure 1. Verification and distribution of pre-mRNA splicing events. (**a**) Verification of novel pre-mRNA splicing events in ORF46 and K10 by PCR. The fragments amplified from unspliced transcripts and spliced transcripts are indicated with orange and blue arrows, respectively (representative of three independent experiments). (**b**) Pie chart depiction of the percentage of identified splicing events happened in clustered viral genes sharing downstream poly(A) site. Source data are provided as a Source Data file.

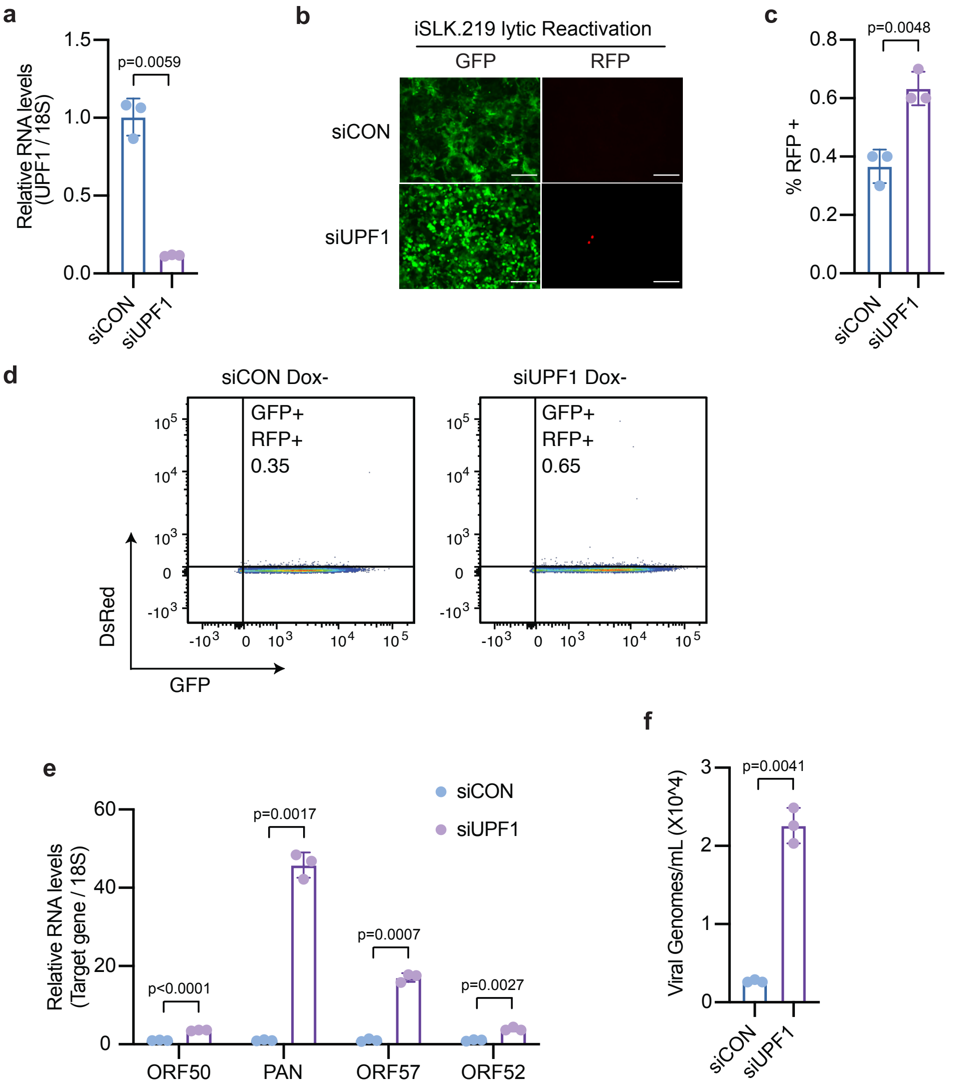

Supplementary Figure 2. Knockdown of UPF1 enhances KSHV spontaneous lytic reactivation in iSLK.219 cells. (**a**) UPF1 knockdown efficiency determined by RT-qPCR. (**b**) GFP and RFP in iSLK.219 cells were imaged 96 h post-siRNA transfection (representative of three independent experiments). Bar indicates 250 μm. (**c**) RFP positive cells in (b) were quantified by flow cytometry. (**d**) The representative flow cytometry plot for (c). (**e**) Quantification of indicated gene expression by RT-qPCR 72 h post-siRNA transfection. (**f**) Quantification of virion production in the supernatant of iSLK.219 cells by qPCR. Data are presented as mean values +/- SD（n=3 biologically independent samples). p-values were determined by the two-tailed Student's t-test. Source data are provided as a Source Data file.

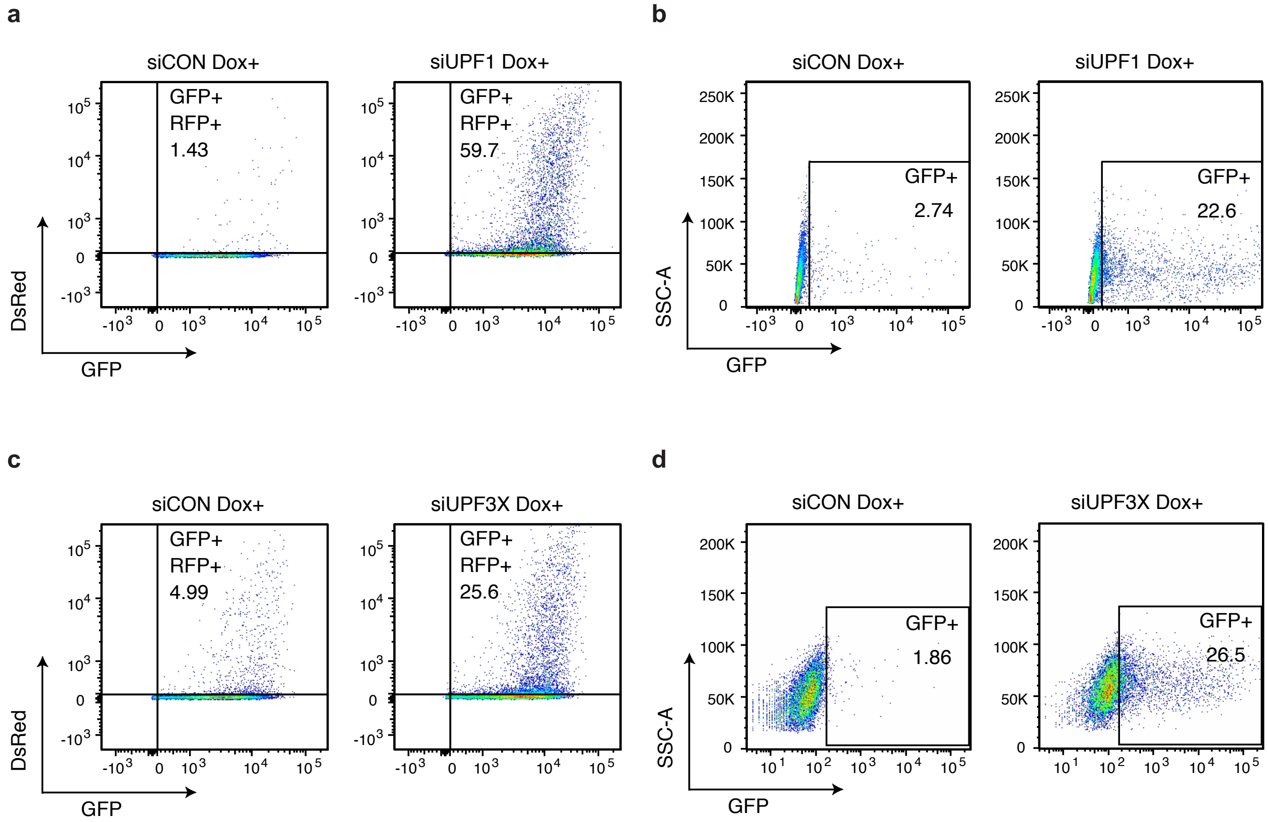

Supplementary Figure 3. The representative flow cytometry plots for Figure 2. (**a**) Figure 2d; (**b**) Figure 2h; (**c**) Figure 2l; (**d**) Figure 2p.

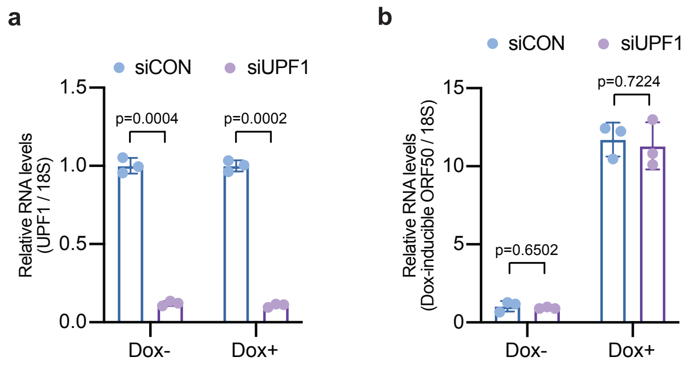

Supplementary Figure 4. Knockdown of UPF1 does not affect Dox-induced RTA expression in uninfected iSLK cells. (**a**) UPF1 knockdown efficiency determined by RT-qPCR. (**b**) RTA expression quantified by RT-qPCR in uninfected iSLK cells 24 h post-Dox treatment. Data are presented as mean values +/- SD (n=3 biologically independent samples). p-values were determined by the two-tailed Student's t-test. Source data are provided as a Source Data file.

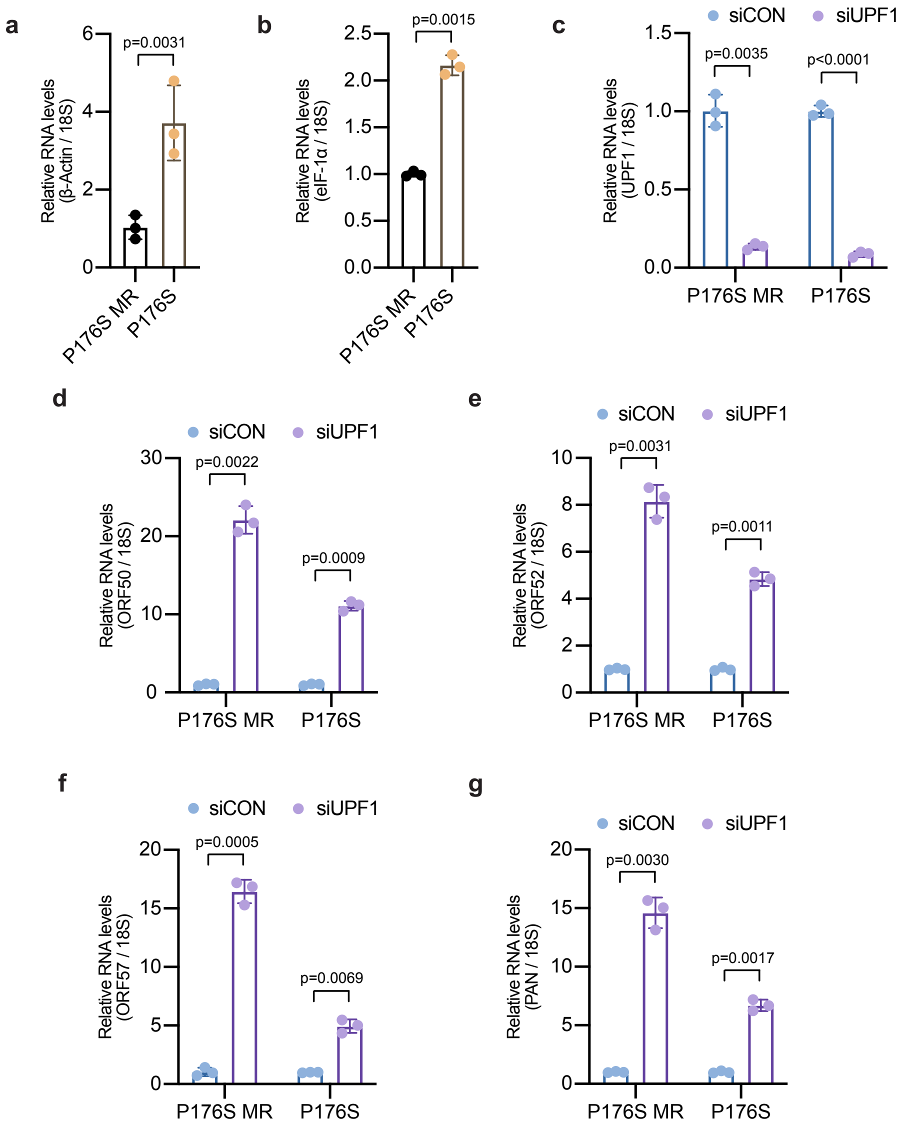

Supplementary Figure 5. Knockdown of UPF1 enhances the lytic reactivation of Sox mutant virus. (**a and b**) Quantification of Sox targeted genes, β-actin (a) and eIF-1α (b), in Sox mutant (P176S) and Sox mutant rescued (P176S MR) cells by RT-qPCR. (**c**) Quantification of UPF1 knockdown efficiency in Sox mutant (P176S) and Sox mutant rescued (P176S MR) cells by RT-qPCR. (**d-g**) Quantification of viral lytic gene expression in Sox mutant (P176S) and Sox mutant rescued (P176S MR) cells by RT-qPCR. Data are presented as mean values +/- SD (n=3 biologically independent samples). p-values were determined by the two-tailed Student's t-test. Source data are provided as a Source Data file.

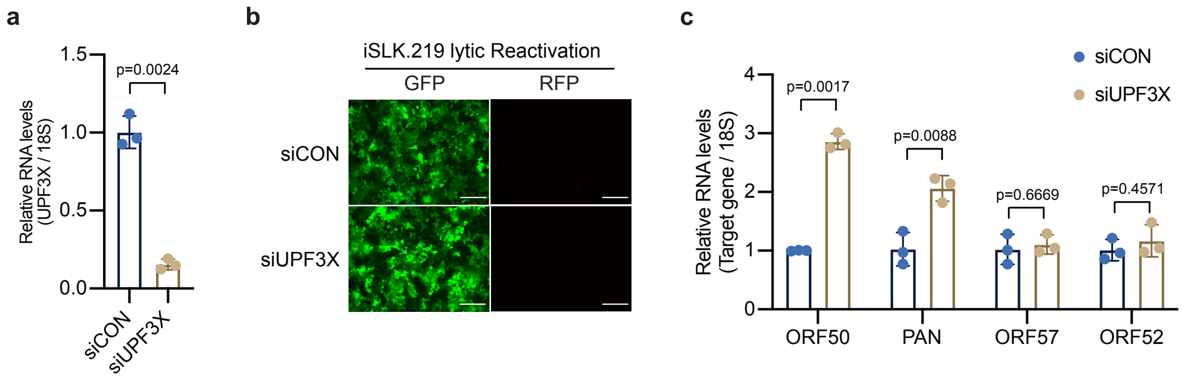

Supplementary Figure 6. Knockdown of UPF3X does not enhance KSHV spontaneous lytic reactivation in iSLK.219 cells. (**a**) UPF3X knockdown efficiency determined by RT-qPCR. (**b**) GFP and RFP in iSLK.219 cells were imaged 96 h post-siRNA transfection (representative of four independent experiments). Bar indicates 250 μm. (**c**) Quantification of indicated gene expression by RT-qPCR in 72 h post-siRNA transfected cells. Data are presented as mean values +/- SD (n=3 biologically independent samples). p-values were determined by the two-tailed Student's t-test. Source data are provided as a Source Data file.

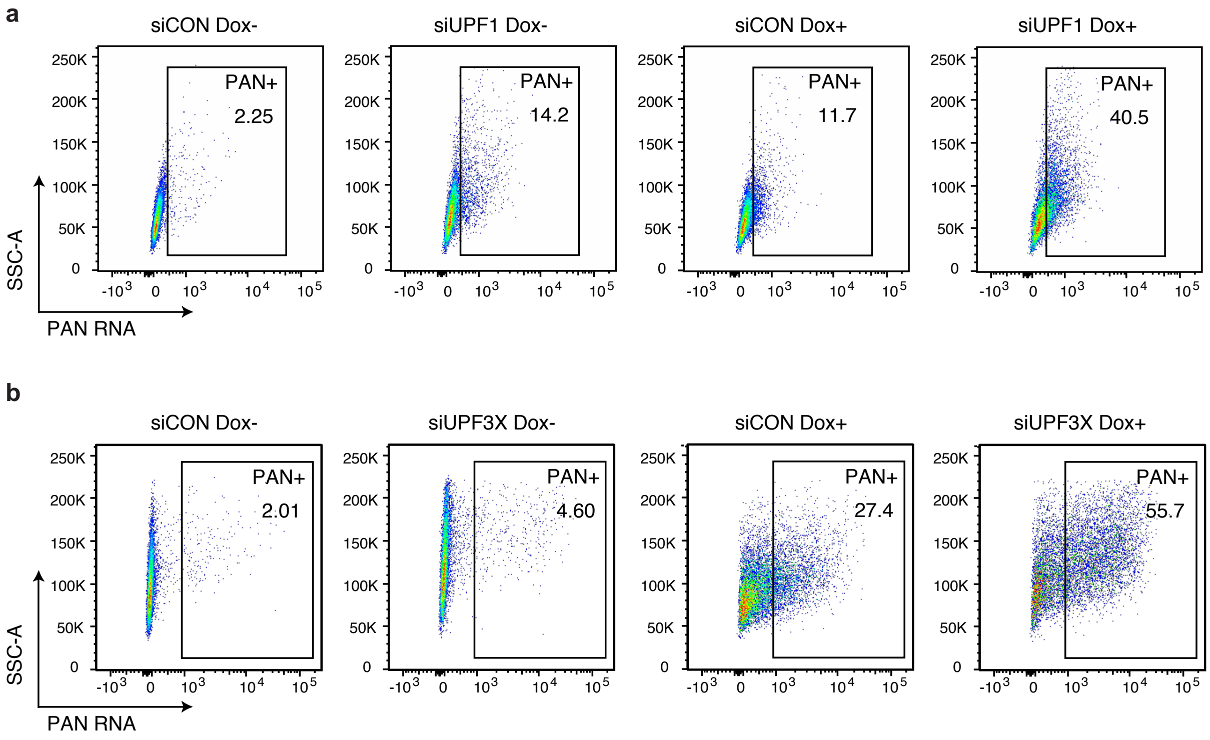

Supplementary Figure 7. The representative flow cytometry plot for Figure 3. (**a**) Figure 3c; (**b**) Figure 3h.

a

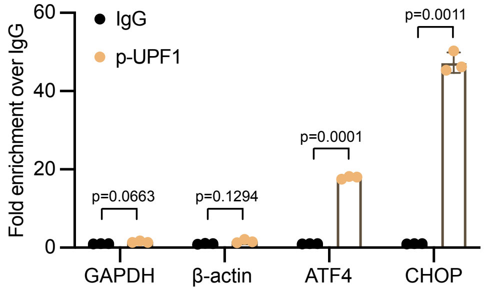

Supplementary Figure 8. Validation of p-UPF1 antibody and immunoprecipitation. (**a**) p-UPF1 fRIP-qPCR analysis of indicated cellular genes in latent TREx-BCBL1-RTA cells. Data are presented as mean values +/- SD（n=3 biologically independent samples). p-values were determined by the two-tailed Student’s t-test. Source data are provided as a Source Data file.

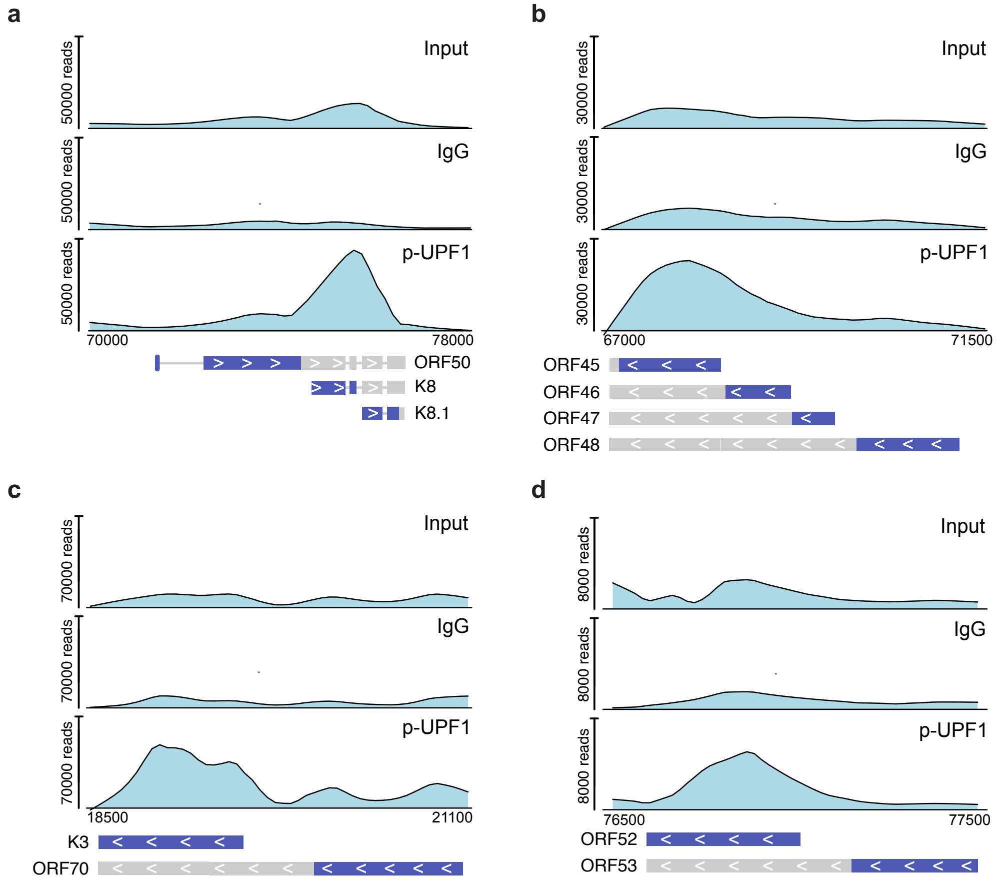

Supplementary Figure 9. Distribution of p-UPF1 fRIP-seq reads on viral gene loci. (**a**) ORF50-K8.1 gene cluster. (**b**) ORF45-ORF48 gene clusters. (**c**) K3-ORF70 gene cluster. (**d**) ORF52-ORF53 gene cluster. A schematic of genomic coordinates of annotated genes is depicted underneath the density plot. The blue bar and the gray bar indicate ORF and 3'UTR respectively.

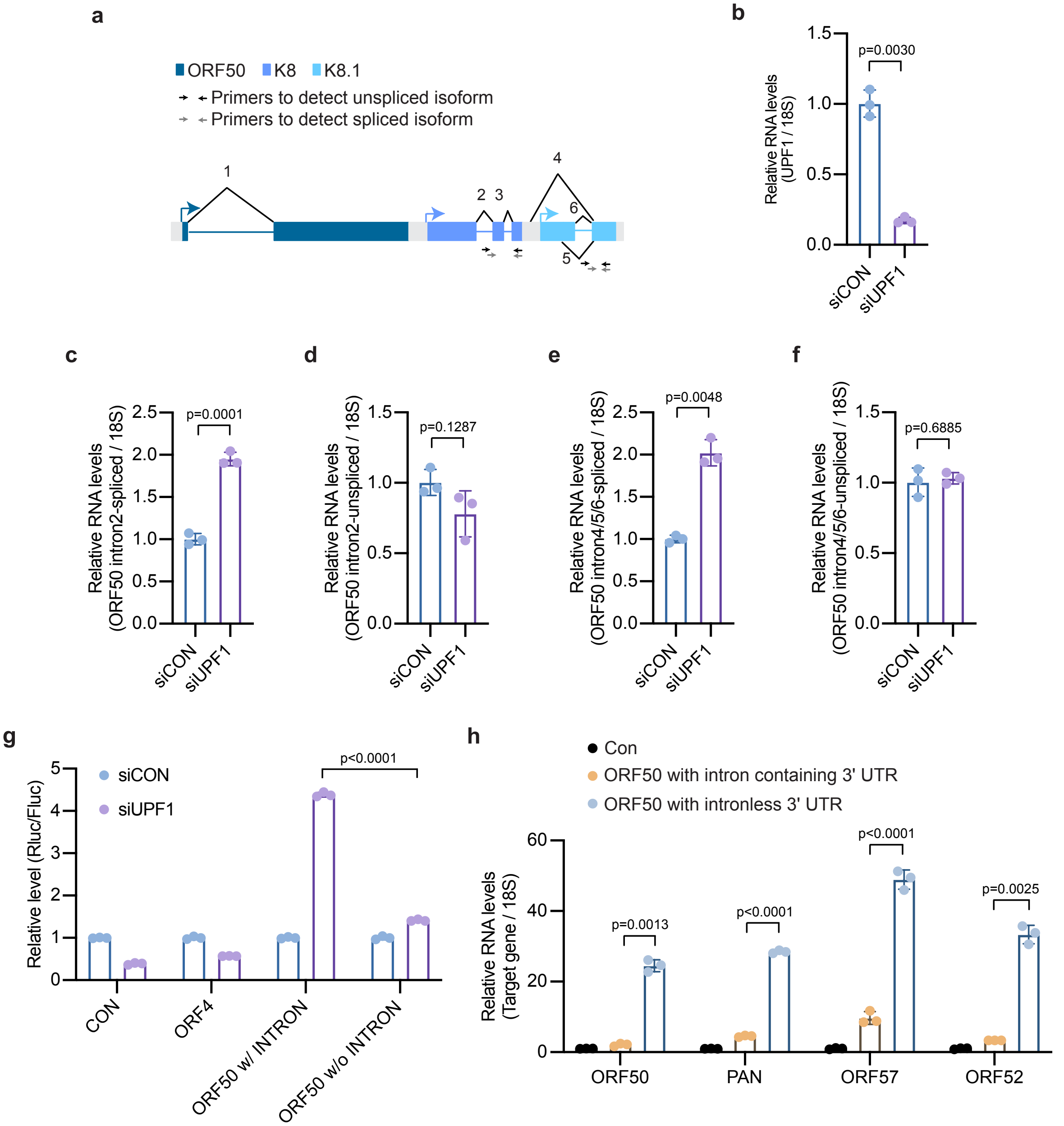

Supplementary Figure 10. Splicing in 3' UTR is sufficient to confer NMD susceptibility of ORF50. (**a**) Schematic ORF50-K8.1 gene cluster. The splicing events are numbered as 1-6. The forward primers to detect unspliced isoforms are designed in the intron (black) and whereas the forward primers to detect spliced isoforms are designed across the intron-exon junction (gray). (**b-f**) HEK293T cells were treated with control or UPF1-targeted siRNA and then transfected with plasmid encoding the full length ORF50 transcript 48 h after siRNA transfection. Quantification of the expression of UPF1 (b), the spliced isoform of ORF50 (c and e) and unspliced isoform of ORF50 (d and f) by RT-qPCR in control (siCON) or UPF1depleted (siUPF1) HEK293T cells. **(g)** Luciferase assay of reporters with control or viral 3'UTR. The ORF50 with intron 3'UTR (ORF50 w/ intron) is cloned from viral genome, whereas the ORF50 without intron 3'UTR (ORF50 w/o intron) is cloned from cDNA. **(h)** TREx-BCBL1-RTA cells are transfected with empty vector (Con), vector encoding ORF50 with intron containing 3' UTR or vector encoding ORF50 with intronless 3' UTR. Cells are collected 24 h after transfection and viral lytic genes are quantified by RT-qPCR. Data are presented as mean values +/- SD (n=3 biologically independent samples). p-values were determined by the two-tailed Student's t-test. Source data are provided as a Source Data file.

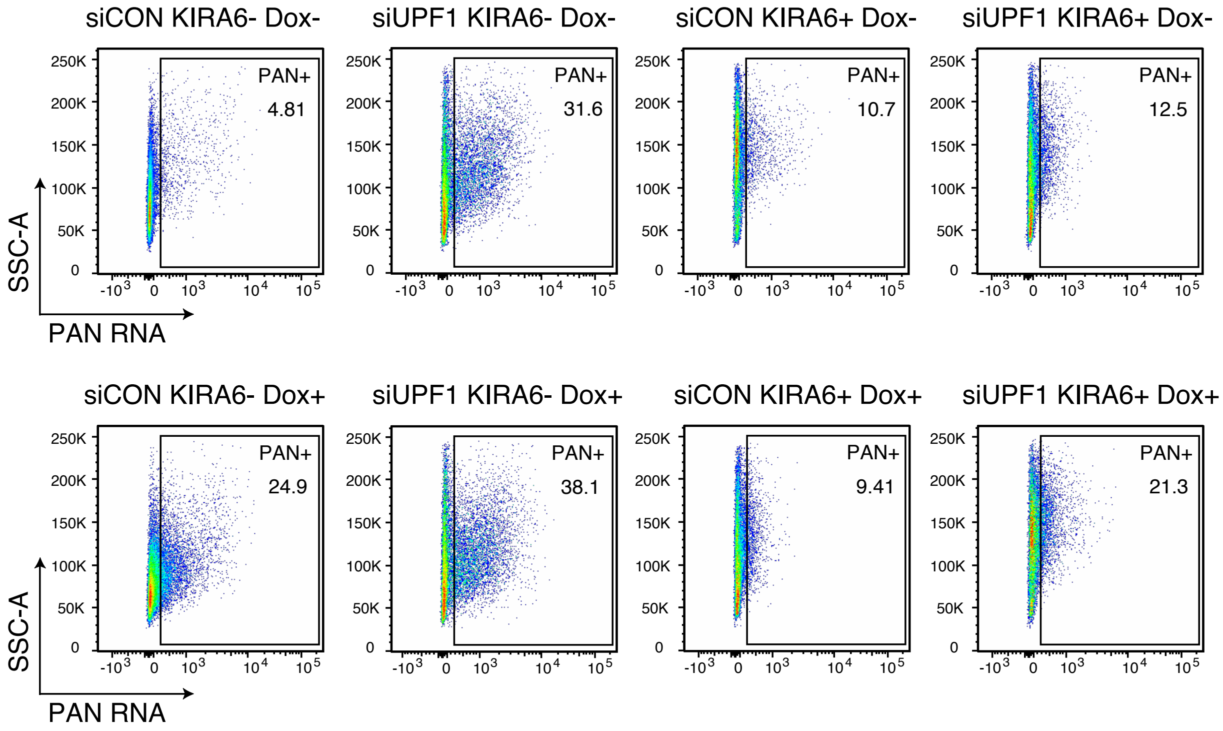

Supplementary Figure 11. The representative flow cytometry plot for Figure 6j.

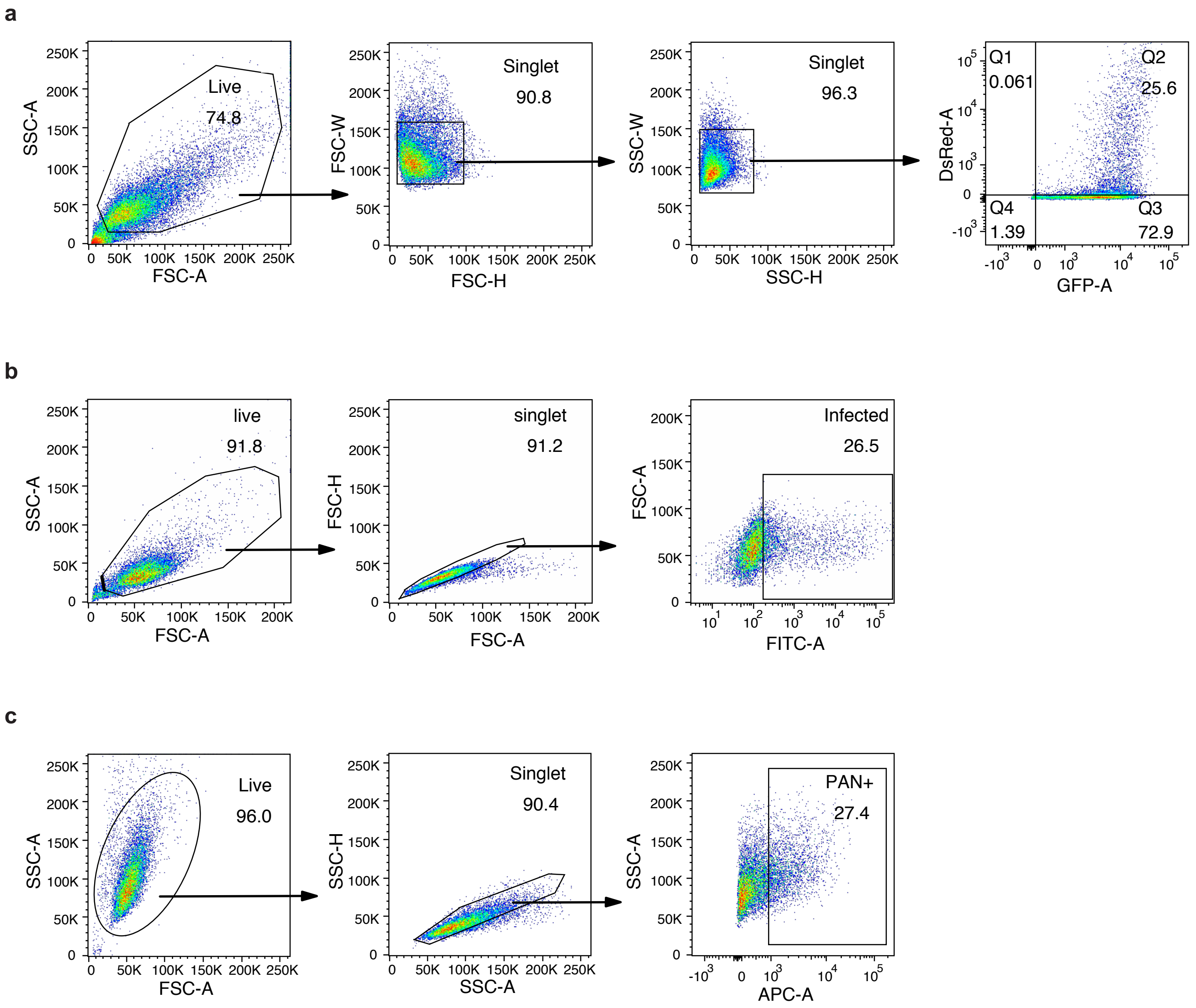

Supplementary Figure 12. Gating strategy used in flow cytometry analysis. (a) Related to Figure 2d, 2l and Supplementary Figure 2d. **(b)** Related to Figure 2h and 2p. **(c)** Related to Figure 3c,3h and 6j.

Supplementary Table 1. List of primers and siRNAs

Oligo and siRNA Name	Sequence 5'-> 3'
ORF57 F	TGGACATTATGAAGGGCATCCTA
ORF57 R	CGGGTTCGGACAATTGCT
PAN F	ATAGGCGACAAAGTGAGGTGGCAT
PAN R	TAACATTGAAAGAGCGCTCCCAGC
ORF52 F	AAATCGAAGCCAGGGTCAGG
ORF52 R	CTCCTCTTCGTCGCCTGTTATTG
ORF50 F	GAGTCCGGCACACTGTACC
ORF50 R	AAACTGCCTGGGAAGTTAACG
K8 F	ATTTCGCAACAGCTTCCAAC
K8 R	TACCTGCTGCAGCTGTCTTG
LANA F	TTGGATCTCGTCTTCCATCC
LANA R	ACCAGACGATGACCCACAAC
vtRNA1-1 F	CGACAGTTCTTTAATTGAAACAAGC
vtRNA1-1 R	AAGGACTGGAGAGCGCCC
18S rRNA F	GTAACCCGTTGAACCCCATT
18SrRNA R	CCATCCAATCGGTAGTAGCG
ORF50 3'UTR F	CCGCTCGAGAGTGTTCGCAAGGGCGTCTG
ORF50 3'UTR R	ATTTGCGGCCGCATGTGAACAATAAACACGTTTATTTACTTTTATGG
ORF48 3UTR F	CCGCTCGAGAAACCGTGTCAGGTAATGCCAC
ORF48 3UTR R	ATTTGCGGCCGCTTATCAGTCCAGCCACGGC
ORF4 3UTR F	CCGCTCGAGGCCTAGACTTGCTCCAGTGTT
ORF4 3UTR R	ATTTGCGGCCGCAATATTAGTTGTCTTGTATTGGTCGG
GADD45A F	GGAGGAATTCTCGGCTGGAG
GADD45A R	CGTTATCGGGGTCGACGTT
ORF48 F	CCAATGTATGACCGTGGTAGAA
ORF48 R	GTCCGAGGTAATGTGCTCTATG
Rluc F	CGCAACTACAACGCCTACCTTC
Rluc R	CCCTCGACAATAGCGTTGGAAAA
ORF46 SP F	CGTGTGTGCTAGACGAGGTC
ORF46 SP R	GAACTTAGGCTAACCACCACAG
K10 SP F	CGTCCATACACCGTCTGTCC
K10 SP R	ATGGCACATGGTAATGGTGC
K10.5 SP F	GTGTACGGCTCGAGAAGCAT
K10.5 SP R	AGAGTCGGCATTGGCATCAT
ORF57 SP F	GTTCCCACGGCCCATTTTTC
ORF57 SP R	GTCGTCCCTCGATTCGTCAA
K8.1 SP F	CTAGCAGCTGGATCTCCGTC
K8.1SP R	CGCGTCTCTTCCTCTAGTCG
ORF50 SP F	CGCAAGGGGTAGTCTGTTGT
ORF50 SP R	AGACCACATAGCGCACCAAG
K3-ORF70 AS F	GACTACTGCCCGCATCCTAC
K3-ORF70 AS R	GACGTTCTCGAGCTCTCCTG
ORF50 Promoter F	CAA AGA GCT TGG GGG GGC AGA
ORF50 Promoter R	TGC CAC CCA GCT ACT GGT TTC
sXBP1 F	CTGAGTCCGAATCAGGTGCAG
sXBP1 R	ATCCATGGGGAGATGTTCTGG
UPF1 F	CCCAAGACTGACTCTGGTAATG
UPF1 R	CCCAACAGCTTGTGGTAGAT
UPF3X F	GAAAGAG CCAGTGG GCAAAGTTG
UPF3X R	CGAAGTA TGCGCTC CTGA TCT C
IRE1α F	TGCAGGTCCCAACACATGTGG
IRE1α R	TCAGGCCTTCATTATTCTTGC
CHOP F	AGAACCAGGAAACGGAAACAGA

CHOP R	TCTCCTTCATGCGCTGCTTT
siUPF1 sense	GAUGCAGUUCCGCUCCAUU[dT][dT]
siUPF1 antisense	AAUGGAGCGGAACUGCAUC[dT][dT]
siUPF3X sense	GGAGAAGCGAGUAACCCUG[dT][dT]
siUPF3X antisense	CAGGGUUACUCGCUUCUCC[dT][dT]
PAN FISH-1	ACAAATGCCACCTCACTTTGTCGC
PAN FISH-2	CGCTGCTTTCCTTTCACATT
PAN FISH-3	GTGAAGCGGCAGCCAAGGTGACTGG
ORF50 3UTR SP1 F	TGCGTCTGTAGTTAAGGCCGAAG
ORF50 3UTR SP1 R	TCCTTATGTGCCTCCAATCTCGC
ORF50 3UTR UNSP1 F	AGCACAGCCCACACATGTC
ORF50 3UTR SP2 F	CCAGACTTTGTGTGGATCATATTCATC
ORF50 3UTR SP2 R	CTCTAGTCGTTGTAGTGGTGGC
ORF50 3UTR UNSP2 F	CCTTACGTTCTGTCTCATCTACAGG
