# Supplementary material for: The RNA quality control pathway nonsense-mediated mRNA decay targets cellular and viral RNAs to restrict KSHV
Source: Nat Commun. 2020 Jul 3;11:3345. doi: 10.1038/s41467-020-17151-2 (PMC7334219; doi:10.1038/s41467-020-17151-2)
Supplement: Supplementary file 3 — Description of Additional Supplementary Files [file 41467_2020_17151_MOESM3_ESM.pdf]

Description of Additional Supplemantary Files

FileName: Supplementary Data 1

Description: Splicing juctions in iSLK.219 cells

FileName: Supplementary Data 2

Description: Splicing juctions in TREx-BCBL1-RTA cells

FileName: Supplementary Data 3

Description: p-UPF1 fRIP enriched genes in latent TREx-BCBL1-RTA cells

FileName: Supplementary Data 4

Description: p-UPF1 fRIP enriched genes in 48hr lytic TREx-BCBL1-RTA cells
