# Supplementary material for: The RNA quality control pathway nonsense-mediated mRNA decay targets cellular and viral RNAs to restrict KSHV
Source: Nat Commun. 2020 Jul 3;11:3345. doi: 10.1038/s41467-020-17151-2 (PMC7334219; doi:10.1038/s41467-020-17151-2)
Supplement: Supplementary file 8 — Reporting Summary [file 41467_2020_17151_MOESM8_ESM.pdf]

nature research

Corresponding author(s): John Karijolich

Last updated by author(s): May 18, 2020

Reporting Summary

Nature Research wishes to improve the reproducibility of the work that we publish. This form provides structure for consistency and transparency in reporting. For further information on Nature Research policies, see Authors & Referees and the Editorial Policy Checklist .

Statistics

For all statistical analyses, confirm that the following items are present in the figure legend, table legend, main text, or Methods section.

n/a	Confirmed	
☐	☒	The exact sample size (*n*) for each experimental group/condition, given as a discrete number and unit of measurement
☐	☒	A statement on whether measurements were taken from distinct samples or whether the same sample was measured repeatedly
☐	☒	The statistical test(s) used AND whether they are one- or two-sided<br>*Only common tests should be described solely by name; describe more complex techniques in the Methods section.*
☒	☐	A description of all covariates tested
☐	☒	A description of any assumptions or corrections, such as tests of normality and adjustment for multiple comparisons
☐	☒	A full description of the statistical parameters including central tendency (e.g. means) or other basic estimates (e.g. regression coefficient) AND variation (e.g. standard deviation) or associated estimates of uncertainty (e.g. confidence intervals)
☐	☒	For null hypothesis testing, the test statistic (e.g. *F*, *t*, *r*) with confidence intervals, effect sizes, degrees of freedom and *P* value noted<br>*Give P values as exact values whenever suitable.*
☒	☐	For Bayesian analysis, information on the choice of priors and Markov chain Monte Carlo settings
☐	☒	For hierarchical and complex designs, identification of the appropriate level for tests and full reporting of outcomes
☐	☒	Estimates of effect sizes (e.g. Cohen's *d*, Pearson's *r*), indicating how they were calculated

Our web collection on statistics for biologists contains articles on many of the points above.

Software and code

Policy information about availability of computer code

Data collection	No software was used.
Data analysis	Flow cytometry data was analyzed with FlowJo X (9.6.4).<br>High throughput sequencing raw reads quality in fastq files were accessed by FastQC (v0.11.8, https://www.bioinformatics.babraham.ac.uk/projects/fastqc/). Reads were aligned to the human reference genome and KSHV genome using STAR (2.6.1b_10-01) under 2-pass mode 80. We estimate the transcript abundance using Rsubread, and enriched transcripts were called using edgeR. Genome coverages were presented with Gviz (1.30.3) or IGV (Linux_2.5.2) viewer. GSEA analysis was performed with R package fgsea (1.10.1, https://bioconductor.org/packages/release/bioc/html/fgsea.html) using hallmark genesets. For evaluation of fRIP reads coverage at 3' end of transcripts, uniquely mapped reads were extracted and transformed to bigwig file using bamCoverage command (deeptools 3.4.3) with the bin size of 10. Then all the transcripts from gencode.v24 annotation was used for coverage analysis using computeMatrix (deeptools: https://deeptools.readthedocs.io/en/develop/content/list_of_tools.html) under reference-point mode (TES) with parameter --metagene set to TRUE. The enrichment of NMD target in UPF1 fRIP-Seq experiment was tested withWilcoxon rank-sum test. kpLogo was used to analysis nucleotide usage at the KSHV 5' and 3' splicing junctions. Statistical analyses were done with R scripts.

For manuscripts utilizing custom algorithms or software that are central to the research but not yet described in published literature, software must be made available to editors/reviewers. We strongly encourage code deposition in a community repository (e.g. GitHub). See the Nature Research guidelines for submitting code & software for further information.

Data

Policy information about availability of data

All manuscripts must include a data availability statement. This statement should provide the following information, where applicable:

- Accession codes, unique identifiers, or web links for publicly available datasets
- A list of figures that have associated raw data
- A description of any restrictions on data availability

Raw data is provided in the Source Data file.
Sequencing data from this study have been deposited in SRA under project number PRJNA598976 [https://www.ncbi.nlm.nih.gov/Traces/study/?query_key=1&WebEnv=NCID_1_219419849_130.14.22.76_5555_1589812214_3528846834_0MetA0_S_HStore&o=acc_s%3Aa].
A reporting summary for this article is available as a Supplementary file.
Costom code have been deposited to
High throughput sequencing reads were aligned to the human reference genome (gencode GRCh38.p10, https://www.ncbi.nlm.nih.gov/assembly/GCF_000001405.36/) and KSHV genome (GQ994935.1, https://www.ncbi.nlm.nih.gov/nuccore/GQ994935.1).
All the transcripts were summarized to biotypes annotated with GENCODE database (gencode.v24, ftp://ftp.ebi.ac.uk/pub/databases/gencode/Gencode_human/release_24/gencode.v24.chr_patch_hapl_scaff.annotation.gtf.gz).

Field-specific reporting

Please select the one below that is the best fit for your research. If you are not sure, read the appropriate sections before making your selection.

- [x] Life sciences
- [ ] Behavioural & social sciences
- [ ] Ecological, evolutionary & environmental sciences

For a reference copy of the document with all sections, see nature.com/documents/nr-reporting-summary-flat.pdf

Life sciences study design

All studies must disclose on these points even when the disclosure is negative.

Sample size	To perform the statistics and calculate the s.d., at least three biologically independent experiments were performed. The number of biological replicates are indicated in the figure legends
Data exclusions	No data were excluded from the analysis
Replication	All relevant experiments were repeated at least three times. All replication attempts were successful.
Randomization	No randomization was performed, samples were treated according to the same protocols side-by-side with the respective controls
Blinding	Personnel handling the sequencing of fRIP-seq data were blinded. Researchers were blinded to sample identity during imaging and data analysis.

Reporting for specific materials, systems and methods

We require information from authors about some types of materials, experimental systems and methods used in many studies. Here, indicate whether each material, system or method listed is relevant to your study. If you are not sure if a list item applies to your research, read the appropriate section before selecting a response.

Materials & experimental systems

n/a	Involved in the study	
[ ]	[x]	Antibodies
[ ]	[x]	Eukaryotic cell lines
[x]	[ ]	Palaeontology
[x]	[ ]	Animals and other organisms
[x]	[ ]	Human research participants
[x]	[ ]	Clinical data

Methods

n/a	Involved in the study	
[x]	[ ]	ChIP-seq
[ ]	[x]	Flow cytometry
[x]	[ ]	MRI-based neuroimaging

Antibodies

Antibodies used	Antibodies: UPF1 (Abcam, #ab109363, 1:10000), p-UPF1 (Ser1127, EMD Millipore, #07-1016, 1:1000), eIF4A3 (Bethyl, A302-981A, 1:5000), GAPDH (Invitrogen, GA1R, #MA5-15738, diluted 1:5000), β-actin (Invitrogen, BA3R, #MA5-15739, 1:1000), ORF50 and ORF59 (1:10,000, homemade antibody kindly provided by Dr. Britt Glaunsinger, University of California, Berkeley),

	bZIP (1:2000, homemade antibody kindly provided by Dr. Cyprian Rossetto, University of Nevada, Reno), FLAG (Thermo Fisher Scientific, FG4R, MA1-91878, 1:1000), XBP1 (Abcam, ab220783, 1:1000). Primary antibodies were followed by AlexaFluor 680-conjugated secondary antibodies (Life Technologies, goat anti-rabbit #A27042, goat anti-mouse #A28183, 1:10,000)
Validation	UPF1 (abcam), p-UPF-1 (EMD Millipore), eIF4A3 (Bethyl), GAPDH (Invitrogen) B-actin (Invitrogen) (FLAG) Thermo), XBP1 (Abcam) were validated on the manufacturer website for western blot, IP, and immunofluorescence.<br>anti-ORF50 and anti-ORF59 were validated in Hesser et al. PLoS Pathog. 2018 Apr 16;14(4):e1006995. doi: 10.1371/journal.ppat.1006995.<br>anti-bZIP was validated in Zhao et al. Nature Communications volume 9, Article number: 4841 (2018).

Eukaryotic cell lines

Policy information about cell lines

Cell line source(s)	TREx-BCBL1-RTA, iSLK.219 were provided by Dr. Britt Glaunsinger (UC Berkeley). The iSLK.219 cell line was generated by Myoung and Gamen, J Virol Methods. 2011 Jun; 174(1-2): 12–21. The TREx-BCBL1-RTA cell line was generated by Nakamura et al. J Virol. 2003 Apr; 77(7): 4205–4220.<br>HEK293T cells were purchased from ATCC.
Authentication	Cell lines were obtained from investigators who generated them, and then passed through an intermediary, or generated in our lab. They are not further authenticated.
Mycoplasma contamination	All cell lines used in this study were mycoplasma free. Cell lines were routinely tested for mycoplasma by PCR.
Commonly misidentified lines<br>(See ICLAC register)	No commonly misidentified cell lines were used in the study.

Flow Cytometry

Plots

Confirm that:

☒ The axis labels state the marker and fluorochrome used (e.g. CD4-FITC).

☒ The axis scales are clearly visible. Include numbers along axes only for bottom left plot of group (a 'group' is an analysis of identical markers).

☒ All plots are contour plots with outliers or pseudocolor plots.

☒ A numerical value for number of cells or percentage (with statistics) is provided.

Methodology

Sample preparation	TREx-BCBL1-RTA cells were fixed in 4% (vol/vol) paraformaldehyde for 30 min at RT, washed with PBS-FISH buffer (1XPBS, 0.2 mg/ml RNase-free BSA) twice, and then permeabilized with 1X PBS containing 0.2% (vol/vol) Tween-20 for another 30 min at RT. The permeabilized cells were then hybridized with Alexa-Fluor 488 or Alexa-Fluor 647 labeled PAN anti-sense oligos (sequences in Supplementary Table 5) in HB 10% dx buffer (10% (wt/vol) dextran sulfate, 2× saline-sodium citrate (SSC), 10% (vol/vol) formamide, 1 mg/ml tRNA and 0.2 mg/ml BSA) at 37 °C overnight. After extensive washing with HBW buffer (2× SSC, 10% (vol/vol) formamide and 0.2 mg/ml RNase-free BSA), cells were analyzed
Instrument	iSLK.219 cell flow cytometry analysis was performed on a BD LSR Fortessa instrument and HEK293T cell flow cytometry analysis and TREx-BCBL1-RTA cell FISH-FLOW was performed on a BD Canto II instrument.
Software	FlowJo X software
Cell population abundance	Not applicable
Gating strategy	For iSLK.219 cells we first gated FSC-A and SSC-A. After that, we gated on single cells and then gated on GFP and RFP positive cells. For HEK293T cells we first gated FSC-A and SSC-A. After that, we gated on single cells and the GFP positive cells. For TREx-BCBL1-RTA cells, we first gated FSC-A and SSC-A. After that, we gated on single cells and then gated on FITC or APC.

☒ Tick this box to confirm that a figure exemplifying the gating strategy is provided in the Supplementary Information.
